# A Modified Nonlinear Damage Accumulation Model for Fatigue Life Prediction Considering Load Interaction Effects

**DOI:** 10.1155/2014/164378

**Published:** 2014-01-20

**Authors:** Huiying Gao, Hong-Zhong Huang, Shun-Peng Zhu, Yan-Feng Li, Rong Yuan

**Affiliations:** School of Mechanical, Electronic, and Industrial Engineering, University of Electronic Science and Technology of China, No. 2006, Xiyuan Avenue, West Hi-Tech Zone, Chengdu, Sichuan 611731, China

## Abstract

Many structures are subjected to variable amplitude loading in engineering practice. The foundation of fatigue life prediction under variable amplitude loading is how to deal with the fatigue damage accumulation. A nonlinear fatigue damage accumulation model to consider the effects of load sequences was proposed in earlier literature, but the model cannot consider the load interaction effects, and sometimes it makes a major error. A modified nonlinear damage accumulation model is proposed in this paper to account for the load interaction effects. Experimental data of two metallic materials are used to validate the proposed model. The agreement between the model prediction and experimental data is observed, and the predictions by proposed model are more possibly in accordance with experimental data than that by primary model and Miner's rule. Comparison between the predicted cumulative damage by the proposed model and an existing model shows that the proposed model predictions can meet the accuracy requirement of the engineering project and it can be used to predict the fatigue life of welded aluminum alloy joint of Electric Multiple Units (EMU); meanwhile, the accuracy of approximation can be obtained from the proposed model though more simple computing process and less material parameters calling for extensive testing than the existing model.

## 1. Introduction

Many mechanical components experiencing cyclic loading with variable amplitude are prone to fail due to fatigue; thus, fatigue life prediction of these mechanical components has become a focal research issue. Fatigue life prediction subjected to variable amplitude loading is a complex problem in engineering practices. Compared to constant amplitude loading, it is much more intractable to deal with this sort of problem. Among the problems of fatigue life prediction, one of the most important and rudimentary ones is the modeling of fatigue damage accumulation [1].

Currently, the models used to describe fatigue damage accumulation can be classified into two categories: the linear and nonlinear approaches. Palmgren-Miner rule (just the Miner's rule for short) is the epitome of linear damage accumulation approach and receives extensive usage in engineering machinery due to its simplicity [2]. The drawback of the Miner's rule is the hypothesis that damage accumulation has nothing to do with the load conditions, the load sequences, the interaction between various loads, and the damage induced by stresses below the fatigue limit [3]. To remedy the drawback of the Miner's rule, many fatigue damage accumulation methods have been proposed and a majority of these models are based on nonlinear accumulation laws.

The nonlinear fatigue damage accumulation models can be classified into the following categories: damage curve based approaches [4], continuum damage mechanics models [5–8], interaction between various loads considered models [9–11], energy based methods [12–16], physical properties degradation based model [14, 17, 18], ductility exhaustion based methods [19, 20], and thermodynamic entropy based theories [21, 22]. Detailed comments on these models can be found in [23].

In general, load sequences and interaction effects are two important issues in the fatigue damage accumulation. A nonlinear fatigue damage accumulation model which is on the basis of damage curve approach explains the influence of load sequences very well, but there is little illustration about load interaction effects. The purpose of this paper is to propose a modified nonlinear fatigue damage accumulation model based on damage curve approach to consider the load interaction effects. The structure of this paper is organized as follows. A nonlinear fatigue damage accumulation model proposed by Manson and Halford [4] is briefly introduced and the comparison between predicted results through this model and experimental data of two metallic materials is made to provide a fundamental basis for proposing a modified model. Then, the same sets of experimental data are used to validate the proposed model under two-level load conditions. Finally, comparison analysis of predictions by the proposed model and two existing models is carried out for further validating the accuracy of the modified model.

## 2. Nonlinear Fatigue Damage Accumulation Model Based on Damage Curve Approach

### 2.1. Nonlinear Fatigue Damage Accumulation Model Based on Damage Curve Approach

Early in 1954, Marco and Starkey [24] proposed a nonlinear fatigue damage accumulation model; subsequently, a lot of research work had been carried out and the approaches also were continuously being improved. A damage accumulation model based on damage curve approach proposed by Manson and Halford (just the Manson-Halford model for short) is investigated in this section; the effects of load sequences under two-level loading are explained very well by this model. The detailed derivation process can be found in [4]; only a brief introduction is given in this section.

Manson and Halford obtained the expression of crack length through detailed deducing, which can be expressed by

(1) $$\begin{array}{c} a=a_{0}+\left(0.18-a_{0}\right){\left(\frac{n_{a}}{N_{f}}\right)}^{(2/3)N_{f}^{0.4}}. \end{array}$$

Then, the damage is given as a function of crack length *a*_0_ and cycle ratio

(2) $$\begin{array}{c} D=\frac{1}{0.18}\left[a_{0}+\left(0.18-a_{0}\right){\left(\frac{n_{a}}{N_{f}}\right)}^{(2/3)N_{f}^{0.4}}\right], \end{array}$$

where *n*_*a*_ is the number of cycles to reach a crack length of *a*, *N*_*f*_ is the number of cycles to failure, and *a*_0_ is the characteristic defect length of the material when *n*_*a*_/*N*_*f*_ = 0.

The nonlinear fatigue damage accumulation model under multilevel loading can be described as follows. Firstly, suppose if a loading stress *σ*_1_ is applied to the material for *n*_1_ cycles and the damage increases from 0 to point *A* along with a damage curve Γ_1_, then a different loading stress *σ*_2_ is applied for *n*_2_ cycles and the damage will increase from point *B* to *C* along with another damage curve Γ_2_. Using (2), the damage at points *A* and *B* can be obtained as

(3) $$\begin{array}{c} D_{A}=\frac{1}{0.18}\left[a_{0}+\left(0.18-a_{0}\right){\left(\frac{n_{1}}{N_{f1}}\right)}^{(2/3)N_{f}^{0.4}}\right],\\ D_{B}=\frac{1}{0.18}\left[a_{0}+\left(0.18-a_{0}\right){\left(\frac{n_{2}^{\prime }}{N_{f2}}\right)}^{(2/3)N_{f}^{0.4}}\right]. \end{array}$$

According to the damage characteristic of materials, the accumulated damage at point *A* is equal to that at point *B*. Through equating (3), the following expression can be obtained; that is, to make the damage increase from 0 to point *B* along with the damage curve Γ_2_, the following cycle ratio is needed:

(4) $$\begin{array}{c} \frac{n_{2}^{\prime }}{N_{f2}}={\left(\frac{n_{1}}{N_{f1}}\right)}^{{\left(N_{f1}/N_{f2}\right)}^{0.4}}, \end{array}$$

and the cycle ratio after the loading cycles at *σ*_2_ becomes

(5) $$\begin{array}{c} \frac{n_{2}^{\prime }}{N_{f2}}+\frac{n_{2}}{N_{f2}}={\left(\frac{n_{1}}{N_{f1}}\right)}^{{\left(N_{f1}/N_{f2}\right)}^{0.4}}+\frac{n_{2}}{N_{f2}}. \end{array}$$

Similarly, the total cycle ratio after the action of the loading stress *σ*_3_ for *n*_3_ cycles is obtained; that is,

(6) $$\begin{array}{c} {\left\{{\left(\frac{n_{1}}{N_{f1}}\right)}^{{\left(N_{f1}/N_{f2}\right)}^{0.4}}+\frac{n_{2}}{N_{f2}}\right\}}^{{\left(N_{f2}/N_{f3}\right)}^{0.4}}+\frac{n_{3}}{N_{f3}}. \end{array}$$

Assuming that the sum of (6) is equal to unity, fatigue failure occurs. By analogy, the damage accumulation rule under multilevel loading can be described as

(7) [[[(n1Nf1)α1,2+n2Nf2]α2,3+n3Nf3]α3,4+⋯+ni−1Nf(i−1)]αi−1,i  +niNfi=1,

where

(8) $$\begin{array}{c} \alpha_{i-1,i}={\left(\frac{N_{f(i-1)}}{N_{fi}}\right)}^{0.4}, \end{array}$$

where the subscripts 1,2, 3,…, *i* − 1, *i* are the sequence numbers of loading stress, *n*_1_, *n*_2_, *n*_3_, …, *n*_*i*−1_, *n*_*i*_ are the cycle numbers under different loading stress, and *N*_*f*1_, *N*_*f*2_, *N*_*f*3_, …, *N*_*f*(*i*−1)_, *N*_*fi*_ represent the fatigue life under *σ*_1_, *σ*_2_, *σ*_3_, …, *σ*_*i*−1_, *σ*_*i*_, respectively.

When the loading stress applied at all levels is equal to each other, *N*_*f*1_ = *N*_*f*2_ = *N*_*f*3_ = ⋯ = *N*_*f*(*i*−1)_ = *N*_*fi*_; thus, *α*_1,2_ = *α*_2,3_ = *α*_3,4_ = ⋯ = *α*_*i*−1,*i*_ = 1; therefore, (7) and (8) can be simplified into the Miner's rule.

For Manson-Halford model, the exponent parameter *α* is a key factor which can produce more accurate results if *α* is determined adequately. The detailed deterministic process of exponent parameter *α* and the material constant 0.4 was given by Manson and Halford based on the concept of effective microcosmic crack growth [4].

### 2.2. Comparison of Experimental Data and the Model Prediction Results

In this section, the predicted damage results of two different materials are obtained using Manson-Halford model. Two materials, that is, 45 and 16Mn steels, respectively, are used and tests are carried out under two-level loading, that is, high-low and low-high loading. For 45 steel, the high-low loading spectrum is 331.46–284.4 Mpa, while the low-high loading spectrum is 284.4–331.46 Mpa. For 16Mn steel, the high-low and low-high loading spectra are 562.9–392.3 Mpa and 372.65–392.3 Mpa, respectively. More details can be found in [25–27].

According to (7) and (8), the damage accumulation model under two-level loading can be expressed as

(9) $$\begin{array}{c} {\left(\frac{n_{1}}{N_{f1}}\right)}^{\alpha }+\frac{n_{2}}{N_{f2}}=1 \end{array}$$

or

(10) $$\begin{array}{c} \frac{n_{2}}{N_{f2}}=1-{\left(\frac{n_{1}}{N_{f1}}\right)}^{\alpha },\\ \alpha ={\left(\frac{N_{f1}}{N_{f2}}\right)}^{0.4}, \end{array}$$

where *n*_1_, *n*_2_ indicate the number of cycles at the 1st and 2nd loading stress levels, and *N*_*f*1_ and *N*_*f*2_ represent the fatigue failure life at the corresponding load levels, respectively.

Thus, the cycle ratio predictions at the 2nd loading stress level can be obtained from (9) and (10). The experimental data and the model prediction results are listed in Tables 1 and 2. The abbreviations in these two tables are explained as follows: the “E.” and “P.” represent experimental and model prediction results, respectively.

**Figure 1:**
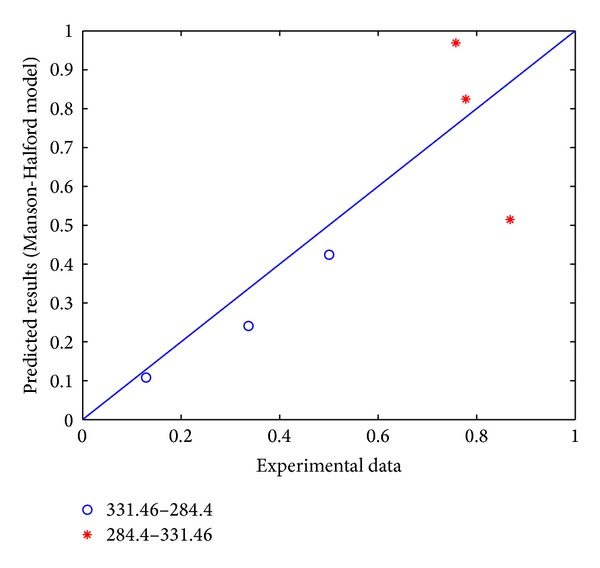
Comparison of Manson-Halford model prediction results and experimental data for 45 steel.

**Figure 2:**
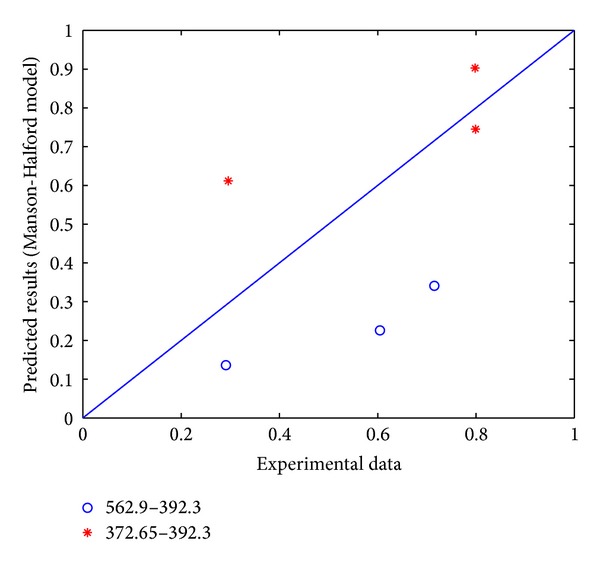
Comparison of Manson-Halford model prediction results and experimental data for 16Mn steel.

Comparing the experimental data with the model prediction results of Manson-Halford model, it is obvious that most predicted results are close to the experimental data. As shown in Figures 1 and 2, it should be noted that they are relatively close to practical situation. It needs to be pointed out that in this paper, the “*A*-*B*”-shaped format in the figures indicates that the loading stress level changes from “*A* Mpa” to “*B* Mpa.”

## 3. A Modified Nonlinear Fatigue Damage Accumulation Model Considering the Load Interaction Effects

As shown in Tables 1 and 2, the Manson-Halford model prediction results are relatively close to the experimental data. However, it should be noted that there is a large difference between the experimental data and predicted value for 16Mn steel. Meanwhile, under high-low loading conditions, the predicted results are lower than experiment data, and most predictions are larger than practical value under low-high loading conditions. This may be caused by considering load sequences only without laying enough emphasis on the influence of load interaction. Therefore, Manson-Halford model will be modified in this paper to consider the load interaction effects and aforementioned problems.

Based on Manson-Halford model, Xu et al. [28] suggested that the exponent parameter *α*_*i*−1,*i*_ should be modified to include load amplitude and effective stress related to loading path, whereas in (7)-(8), only the effects of load sequences had been taken into account. Moreover, in view of the current situation that some existing models, such as Corten-Dolan model and Freudenthal-Heller model, are in the form of load amplitude ratio to consider the load interaction effects [10, 29], hence refer to the models in [10, 28, 29], to consider the effects of load interaction and error distribution, the exponent parameter *α*_*i*−1,*i*_ can be modified as

(11) $$\begin{array}{c} \alpha_{i-1,i}={\left(\frac{N_{f(i-1)}}{N_{fi}}\right)}^{0.4\cdot \min\left\{\sigma_{i-1}/\sigma_{i},\sigma_{i}/\sigma_{i-1}\right\}}. \end{array}$$

Then the damage accumulation model under two-level loading can be described as

(12) $$\begin{array}{c} {\left(\frac{n_{1}}{N_{f1}}\right)}^{\alpha }+\frac{n_{2}}{N_{f2}}=1, \end{array}$$

where

(13) $$\begin{array}{c} \alpha ={\left(\frac{N_{f1}}{N_{f2}}\right)}^{0.4\cdot \min\left\{\sigma_{1}/\sigma_{2},\sigma_{2}/\sigma_{1}\right\}}. \end{array}$$

For high-low loading conditions, 0 < *N*_*f*1_/*N*_*f*2_ < 1; then 0 < *α* < 1; therefore, the damage caused by the 2nd loading stress level should meet the following expression:

(14) $$\begin{array}{c} \frac{n_{2}}{N_{f2}}=1-{\left(\frac{n_{1}}{N_{f1}}\right)}^{\alpha }<1-\frac{n_{1}}{N_{f1}}. \end{array}$$

Hence, the cumulative damage under high-low loading conditions is

(15) $$\begin{array}{c} \frac{n_{1}}{N_{f1}}+\frac{n_{2}}{N_{f2}}=\frac{n_{1}}{N_{f1}}+1-{\left(\frac{n_{1}}{N_{f1}}\right)}^{\alpha }<1. \end{array}$$

Similarly, for low-high loading conditions, *N*_*f*1_/*N*_*f*2_ > 1, *α* > 1; then *n*_2_/*N*_*f*2_ = 1 − (*n*_1_/*N*_*f*1_)^*α*^ > 1 − (*n*_1_/*N*_*f*1_); thus, the cumulative damage under low-high loading conditions is

(16) $$\begin{array}{c} \frac{n_{1}}{N_{f1}}+\frac{n_{2}}{N_{f2}}=\frac{n_{1}}{N_{f1}}+1-{\left(\frac{n_{1}}{N_{f1}}\right)}^{\alpha }>1. \end{array}$$

Therefore, the model mentioned above reflects the nonlinearity of damage accumulation and takes the effects of load sequences and load interaction into account simultaneously.

## 4. Validation of the Proposed Model

### 4.1. Validation Study 1

**Figure 3:**
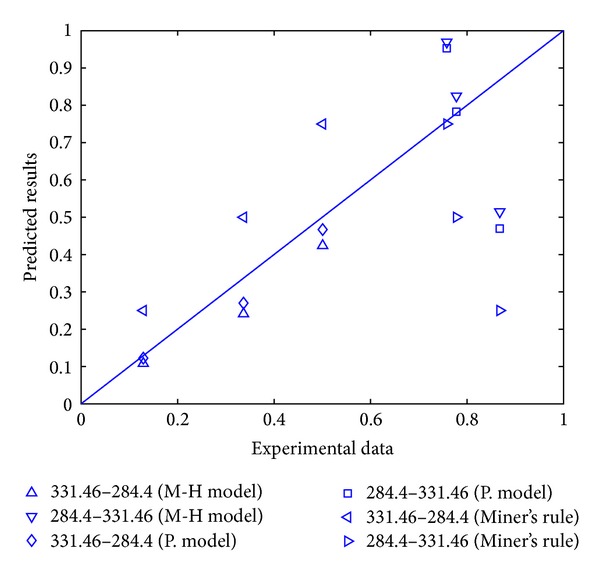
Comparison of prediction results of the proposed model, Miner's rule, and experimental data for 45 steel.

**Figure 4:**
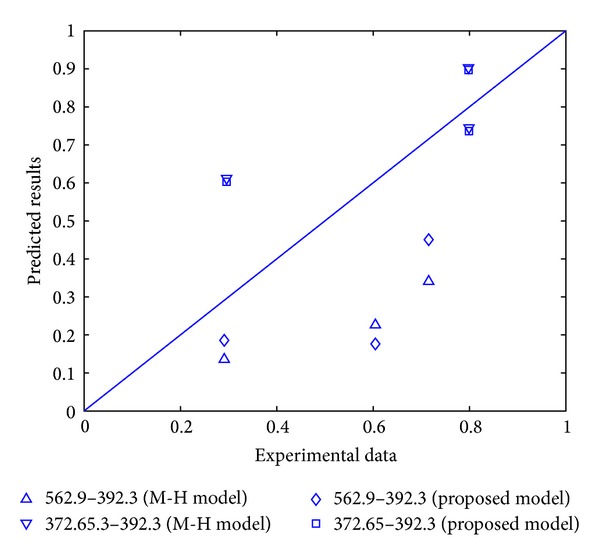
Comparison of prediction results of the proposed model and experimental data for 16Mn steel.

The experimental data adopted here is still the data sets used in Section 2. The comparison of experimental data and the model prediction results by the Manson-Halford model and proposed model can be seen in Figures 3 and 4 (“M-H model” refers to Manson-Halford model and “P. model” represents the proposed model). The results show that nearly 80% of proposed model predictions are better than that by the Manson-Halford model, and the inaccuracy under high-low and low-high loading conditions has been both reduced; this indicates that the predictions by proposed model are more possibly in accordance with the experimental data. Furthermore, Figure 3 shows the predicted results by the Miner's rule for 45 steel; it can be seen that the errors between the prediction results by proposed model and experimental value are smaller than that by Miner's rule. Thus, the proposed model has a better prediction than the Manson-Halford model and Miner's rule.

### 4.2. Validation Study 2

Nowadays, the requirements of high speed are the prospects and development trends of railway passenger, and the high-speed railway also has become an important symbol of modernization of national railway. As a kind of green transportation, the safety and reliability problems of high-speed train are concerned by researchers, although the advantages are well known to everyone. At present, all high-speed passenger car bogie frame and car body have steel and aluminum alloy welded structures, since the self-weight of train structure needs to be considerably reduced, but due to the harsh load conditions and the inherent weld defect such as geometric irregularity nonmetallic inclusion, residual stress, and heat-affected zone (HAZ), welded joints have been turned into the major failure areas of high-speed train structures [30]. Aluminum alloy materials are widely used in the structures of trains, ships, constructions, and so forth because of their low density, high strength, and inoxidability. Therefore, it is of great significance for better fatigue-life prediction of high-speed train and making sure of its safe operation to figure out an appropriate fatigue damage accumulation method for welded aluminum alloy joint.

In the light of the above, experimental data of welded aluminum alloy joint of Electric Multiple Units (EMU) are used in this section to verify the applicability of the proposed model in the fields of high-speed train. There are two sorts of welded aluminum alloy joint used in this section, that is, butt joint and fillet joint, and the tests are also carried out under two-level loading. The experimental data of EMU are listed in Tables 3 and 4. In addition, comparison between the experimental data and predictions by proposed model and an existing model is carried out for further validating the accuracy of the proposed model.

According to the existing model proposed in [31], the fatigue damage curve is

(17) $$\begin{array}{c} D={\left(\frac{n}{N}\right)}^{1+{\left(\text{lo}{\text{g}}_{1/2}(\sigma /\sigma_{s})\right)}^{t}}. \end{array}$$

Hence, the damage accumulation model under two-level loading is given by

(18) $$\begin{array}{c} D=\overset{2}{\underset{i=1}{\sum }}D_{i}={\left[\frac{n_{2}}{N_{f2}}+{\left(\frac{n_{1}}{N_{f1}}\right)}^{a_{1}/a_{2}}\right]}^{a_{2}},\\ a_{1}=1+{\left(\text{lo}{\text{g}}_{1/2}\frac{\sigma_{1}}{\sigma_{s}}\right)}^{t},\\ a_{2}=1+{\left(\text{lo}{\text{g}}_{1/2}\frac{\sigma_{2}}{\sigma_{s}}\right)}^{t}, \end{array}$$

where *D* is the cumulative damage, *a*_*i*_ is the life damage exponent under the *i*th level load, *n*_1_, *n*_2_ and *N*_*f*1_, *N*_*f*2_ represent the same meaning as mentioned above, *σ*_*s*_ refers to material yield strength, and *t* reflects the influence degree of load sequences effects of the specimen, its determination is by means of fitting according to experimental data

As aformentioned, the cumulative damage can be calculated in different way, unlike the modified model. The comparison results of cumulative damage predictions by the existing model and proposed model are shown in Tables 3 and 4.

From Tables 3 and 4, it is obvious that the cumulative damage predictions by the proposed model exceed unity under low-high loading conditions, and when the load amplitude changes from high to low level, the value is less than unity; this verifies the nonlinearity effect of damage accumulation. Moreover, note that the prediction errors of the proposed model are within the range of 20%; this can meet the accuracy requirement of the engineering project, so the proposed model can accurately predict the value of critical damage and it can be used to determine the fatigue-life of welded aluminum alloy joint of Electric Multiple Units (EMU). Furthermore, the prediction results of cumulative damage calculated by these two models are relatively close to each other; thus, the inaccuracy of these two models predictions is also much close. This shows that cumulative damage predictions by the proposed model correspond approximately to the prediction results by the existing model but do not need some precise parameters related to material property and required experimental data regression. Therefore, using the proposed model, fatigue life prediction can be obtained with high-precision, simple computing process and less material parameters than the existing model.

## 5. Conclusion and Discussion

A modified model is presented in this paper for considering load interaction and load sequences effects on the basis of a nonlinear cumulative damage model, that is, Manson-Halford model. The main achievements and conclusions can be summarized as follows.

1. The exponent parameter in Manson-Halford model has been modified to consider the effects of load interaction, which can be characterized by introducing the ratio of applied load amplitude.
2. The experimental data of 45 steel and 16Mn steel are used to validate the accuracy of the modified model through comparing with the predicted results of Manson-Halford model and the proposed model. Through comparative analysis, it is worth noting that the inaccuracy of the proposed model has been reduced not only under high-low loading conditions but also to the contrary, and nearly 80% of proposed model predictions are better than that by Manson-Halford model. On the other hand, the inaccuracy caused by the proposed model is smaller than that by Miner's rule for 45 steel; therefore, fatigue life prediction by the proposed model is more possibly in accordance with the practical situation than Manson-Halford model and Miner's rule.
3. Comparing cumulative damage predictions by the proposed model with the results through an existing model, it can be found that the prediction results of the proposed model can reflect the nonlinearity of damage accumulation, and this proposed model in this paper is applicable to determine the fatigue life of welded aluminum alloy joint of Electric Multiple Units (EMU) because it can be used to accurately predict the value of critical damage. Meanwhile, there is good consistency among these two models; that is, fatigue life prediction can be obtained with high-precision, simple computing process and less material parameters than the existing model.

Although the results are quite close to the experimental data, all validations are carried out under two-level loading, thus there is a requirement for demonstrating the validation under multi-level and random loading conditions.

## Figures and Tables

**Table 1:** Experimental data and the predicted results of 45 steel.

Loading stress level/Mpa	Load sequences	*n*_1_	*n*_1_/*N*_*f*1_	*n*_2_	*n*_2_/*N*_*f*2_ (E.)	*n*_2_/*N*_*f*2_ (P.)	Error/%
331.46–284.4	High-low	12,500	0.250	250,400	0.5008	0.4241	−15.32
		25,000	0.500	168,300	0.3366	0.2411	−28.37
		37,500	0.750	64,500	0.1290	0.1082	−16.12

284.4–331.46	Low-high	12,500	0.250	37,900	0.7580	0.9693	27.88
		250,000	0.500	38,900	0.7780	0.8247	6.00
		375,000	0.750	43,400	0.8680	0.5145	−40.73

**Table 2:** Experimental data and the predicted results of 16Mn steel.

Loading stress level/Mpa	Load sequences	*n*_1_	*n*_1_/*N*_*f*1_	*n*_2_	*n*_2_/*N*_*f*2_ (E.)	*n*_2_/*N*_*f*2_ (P.)	Error/%
562.9–392.3	High-low	1000	0.2520	56,300	0.7154	0.3411	−37.43
		1700	0.4284	476,000	0.6048	0.2263	−62.57
		2450	0.6174	22,900	0.2910	0.1358	−53.33

372.65–392.3	Low-high	64,400	0.240	62,800	0.7980	0.9028	13.13
		116,000	0.433	62,900	0.7990	0.7449	−6.77
		150,000	0.560	23,300	0.2960	0.6118	106.69

**Table 3:** Experimental data and comparison results of cumulative damage predictions by the proposed model and existing model for butt joint.

Load mode	σ_1_/Mpa	σ_2_/Mpa	*n*_1_/10^3^	*n*_2_/10^3^	*N*_*f*1_	*N*_*f*2_	*D* (by the existing model [31])	*D* (by the proposed model)
Mode 1	104	74	109.9	797.6	549,300	1,540,100	0.9260	0.8988
Mode 2	89	74	176.1	1029.2	880,500	1,540,100	1.0810	0.9372
Mode 3	74	89	770.1	545.6	1,540,100	880,500	0.9290	1.0660
Mode 4	74	104	770.1	418.9	1,540,100	549,300	1.0140	1.1053

**Table 4:** Experimental data and comparison results of cumulative damage predictions by the proposed model and existing model for fillet joint.

Load mode	σ_1_/Mpa	σ_2_/Mpa	*n*_1_/10^3^	*n*_2_/10^3^	*N*_*f*1_	*N*_*f*2_	*D* (by the existing model in [31])	*D* (by the proposed model)
Mode 5	93	73	309.9	587.5	619,800	1,546,100	1.0140	0.9056
Mode 6	83	73	476.1	681.1	952,300	1,546,100	1.0270	0.9426
Mode 7	73	83	509.2	708.2	1,546,100	952,300	0.9930	1.0614
Mode 8	73	93	773.0	426.4	1,546,100	619,800	1.0670	1.1029

## References

[1] Ni K, Zhang SK (1999). Fatigue reliability analysis under two stage loading. *ACTA Mechanica Sinica*.

[2] Miner MA (1945). Cumulative damage in fatigue. *Journal of Applied Mechanics*.

[3] Zhu S-P, Huang H-Z, Wang Z-L (2011). Fatigue life estimation considering damaging and strengthening of low amplitude loads under different load sequences using fuzzy sets approach. *International Journal of Damage Mechanics*.

[4] Manson SS, Halford GR (1981). Practical implementation of the double linear damage rule and damage curve approach for treating cumulative fatigue damage. *International Journal of Fracture*.

[5] Chaboche JL, Lesne PM (1988). A non-linear continuous fatigue damage model. *Fatigue and Fracture of Engineering Materials and Structures*.

[6] Cheng G, Plumtree A (1998). A fatigue damage accumulation model based on continuum damage mechanics and ductility exhaustion. *International Journal of Fatigue*.

[7] Dattoma V, Giancane S, Nobile R, Panella FW (2006). Fatigue life prediction under variable loading based on a new non-linear continuum damage mechanics model. *International Journal of Fatigue*.

[8] Besson J (2010). Continuum models of ductile fracture: a review. *International Journal of Damage Mechanics*.

[9] Xu H (1988). *Fatigue Strength*.

[10] Freudenthal AM, Heller RA (1959). On stress interaction in fatigue and cumulative damage rule. *Journal of the Aerospace Science*.

[11] Zhu SP, Huang HZ, Liu Y (2012). A practical method for determining the Corten-Dolan exponent and its application to fatigue life prediction. *International Journal of Turbo and Jet Engines*.

[12] Halford GR (1966). The energy required for fatigue. *Journal of Materials*.

[13] Xiaode N, Guangxia L, Hao L (1987). Hardening law and fatigue damage of a cyclic hardening metal. *Engineering Fracture Mechanics*.

[14] Zhu S-P, Huang H-Z (2010). A generalized frequency separation-strain energy damage function model for low cycle fatigue-creep life prediction. *Fatigue and Fracture of Engineering Materials and Structures*.

[15] Zhu SP, Huang HZ, He LP (2012). A generalized energy-based fatigue-creep damage parameter for life prediction of turbine disk alloys. *Fracture Mechanics*.

[16] Zhu SP, Huang HZ, Ontiveros V, He LP, Modarres M (2012). Probabilistic low cycle fatigue life prediction using an energy-based damage parameter and accounting for model uncertainty. *International Journal of Damage Mechanics*.

[17] Duyi Y, Zhenlin W (2001). A new approach to low-cycle fatigue damage based on exhaustion of static toughness and dissipation of cyclic plastic strain energy during fatigue. *International Journal of Fatigue*.

[18] Zhu S, Huang H, Xie L (2008). Nonlinear fatigue damage cumulative model and the analysis of strength degradation based on the double parameter fatigue criterion. *China Mechanical Engineering*.

[19] Zhu S-P, Huang H-Z, Li H, Sun R, Zuo MJ (2011). A new ductility exhaustion model for high temperature low cycle fatigue life prediction of turbine disk alloys. *International Journal of Turbo and Jet Engines*.

[20] Zhu SP, Huang HZ, Liu Y (2013). An efficient life prediction methodology for low cycle fatigue-creep based on ductility exhaustion theory. *International Journal of Damage Mechanics*.

[21] Risitano A, Risitano G (2010). Cumulative damage evaluation of steel using infrared thermography. *Theoretical and Applied Fracture Mechanics*.

[22] Naderi M, Amiri M, Khonsari MM (2010). On the thermodynamic entropy of fatigue fracture. *Proceedings of the Royal Society A*.

[23] Yang XH, Yao WX, Duan CM (2003). The review of ascertainable fatigue cumulative damage rule. *Engineering Science*.

[24] Marco SM, Starkey WL (1954). A concept of fatigue damage. *Transaction of the ASME*.

[25] Shang DG, Yao WX (1998). Study on nonlinear continuous damage cumulative model for uniaxial fatigue. *Acta Aeronautica Et Astronautica Sinica*.

[26] Ye DY (1996). *Variation in fatigue properties of structural steel and research of new approach for fatigue life prediction [Ph.D. thesis]*.

[27] Shang D-G, Yao W-X (1999). A nonlinear damage cumulative model for uniaxial fatigue. *International Journal of Fatigue*.

[28] Xu J, Shang DG, Sun GQ, Chen H, Liu ET (2012). Fatigue life prediction for GH4169 superalloy under multiaxial variable amplitude loading. *Journal of Beijing University of Technology*.

[29] Corten HT, Dolon TJ Cumulative fatigue damage.

[30] Lu YH (2011). *Study on fatigue reliability of welded bogie frame for raiway vehicle [Ph.D. thesis]*.

[31] Tian J, Liu Z-M, He R (2012). Nonlinear fatigue-cumulative damage model for welded aluminum alloy joint of EMU. *Journal of the China Railway Society*.

